# Unveiling the Potential of *Aloe vera* Gel Supplementation in a Cooling Extender: A Breakthrough in Enhancing Rooster Sperm Quality and Fertility Ability

**DOI:** 10.3390/ani14162290

**Published:** 2024-08-06

**Authors:** Jutarat Pimpa, Supakorn Authaida, Wuttigrai Boonkum, Sarinya Rerkyusuke, Chalinee Janta, Vibuntita Chankitisakul

**Affiliations:** 1 Department of Animal Science, Faculty of Agriculture, Khon Kaen University, Khon Kaen 40002, Thailand; jutarat_p@kkumail.com (J.P.); supakorn.u@kkumail.com (S.A.); wuttbo@kku.ac.th (W.B.); 2 Network Center for Animal Breeding and Omics Research, Khon Kaen University, Khon Kaen 40002, Thailand; 3 Division of Livestock Medicine, Faculty of Veterinary Medicine, Khon Kaen University, Khon Kaen 40002, Thailand; sarinyare@kku.ac.th; 4 Medicinal Plants Innovation Center of Mae Fah Luang University, Chiang Rai 57100, Thailand; chalineejanta14@gmail.com

**Keywords:** antioxidants, chicken, cooled semen, ROS

## Abstract

**Simple Summary:**

Cooling rooster semen to a temperature of 5 °C is a technique utilized to extend the preservation period of sperm. However, rooster sperm is highly susceptible to damage during preservation, which involves numerous sources of oxidative stress from dilution, limiting its viability and fertility potential to less than 24 h under cool storage conditions. Therefore, researchers are continuously working to optimize preservation methods to enhance the viability and longevity of rooster semen. The aim of this study was to investigate the effectiveness of *Aloe vera* gel, known for its beneficial antioxidant properties, on the quality of cooled semen in Thai native roosters (Pradu Hang Dum) and to evaluate the fertility rate after artificial insemination. The results showed that *Aloe vera* gel contained various bioactive compounds with antioxidant activity. The supplementation of the extender with 1.0% *Aloe vera* gel proved to be beneficial, reducing lipid peroxidation and simultaneously improving sperm quality during cold storage at 5 °C for up to 72 h.

**Abstract:**

The cooling of semen storage at 5 °C from a Thai native rooster (Pradu Hang Dum), supplemented with herbs possessing antioxidant properties, provided limited research. This study was conducted to evaluate the efficiency of *Aloe vera* (AV) gel supplementation at various levels on the quality of cooled semen and subsequent fertility after artificial insemination. Sixty-four chickens had semen pooled, diluted, and supplemented with different levels of AV gel (0% as control, 0.25%, 0.50%, 1.0%, 2.5%, 5.0%, 10%, and 20%), and then stored for 72 h. In Experiment 1, semen quality, malondialdehyde (MDA) levels, and pH values were assessed at 0, 24, 48, and 72 h after storage. Experiment 2 assessed fertility potential using the most effective cooled storage semen from Experiment 1. Results showed a decrease in semen quality with prolonged storage time (*p* < 0.001). The highest semen quality was observed in the group supplemented with 1.0% AV gel (*p* < 0.001), whereas the lowest was noted in the 20% AV gel group (*p* < 0.001). Furthermore, the 1.0% AV gel group exhibited the highest semen quality at 24, 48, and 72 h of storage. The evaluation of fertility and hatchability rates revealed a statistically significant improvement in fertility potential (*p* < 0.05) in the group supplemented with 1.0% AV gel. In summary, this study represents the first investigation of stored Thai native rooster semen using a semen extender supplemented with *Aloe vera* gel at 5 °C, demonstrating its efficacy for storage up to 72 h. The addition of 1% AV gel was recommended as an antioxidant supplementation during the semen storage process at 5 °C to enhance semen quality and fertility rates.

## 1. Introduction

Thai native chickens, specifically Pradu Hang Dum, have traditionally been raised for household consumption in backyard farms as a source of additional income in smallholder farms. The meat of native chickens is valued for its firm texture, low fat content, and rich protein content [1,2]. Nevertheless, inadequate nutrition often leads to slower growth rates, extending the time required to reach market weight and making them less competitive compared to commercial broilers. To enhance growth performance and egg production in parent stocks, raising systems have transitioned from backyard setups to utilizing battery cages during the production phase of parent stocks [3]. Concurrently, artificial insemination (AI) has been introduced as a practical method for mating to decrease the number of breeding roosters on farms [4]. Therefore, ensuring the efficiency of collected semen for AI becomes crucial to maximizing fertility potential.

After semen collection, rooster semen is diluted with diluents immediately, not only to increase semen volume but also to prevent sperm death from dehydration [5]. Effective diluents act as an energy source for sperm, enhancing their longevity when preserved as cool semen at 2–5 °C for up to 24 h [6]. Alternatively, if semen is diluted in a saline solution at room temperature, AI should be executed within a few hours after collection [7]. The selection of extenders ought to maintain sperm motility and viability and should have a pH and osmolality similar to that of the seminal fluid of the experimental animals [8]. Despite metabolic limitations at lower temperatures, glycolysis and oxidative phosphorylation processes continue to generate lactic acid, CO_2_, and ATP for sperm function, thereby reducing medium pH and prompting reactive oxygen species (ROS) production during storage [9]. Prolonged storage without ROS scavengers results in oxidative harm and compromised sperm functionality [6]. Hence, developing extenders integrated with antioxidants is imperative to sustain sperm longevity without adverse effects.

*Aloe vera* (AV), scientifically known as *Aloe vera* (L.) Burm. f., is classified under the Asphodelaceae family as a biennial plant. This succulent species encompasses over 300 varieties and features a gel-like substance within its leaves. The inner flesh holds a viscous mucilage resembling a transparent, light-green jelly that turns yellow to brown upon exposure to air. The gel comprises more than 75 ingredients, each potentially contributing through various mechanisms of action either independently or synergistically to elucidate over 200 different constituents. Noteworthy compounds contained within AV gel include mucopolysaccharides, enzymes, sterols, prostaglandins, fatty acids, and amino acids, as well as a diverse array of vitamins and minerals. Active bioactive agents found in *Aloe vera* encompass salicylates, magnesium lactate, acemannan, lupeol, campesterol, β-sitosterol, aloin A, and anthraquinones [10]. Furthermore, *Aloe vera* hosts a minimum of seven superoxide dismutases (SODs) known for their antioxidant properties [11].

Several research studies have highlighted the efficacy of AV gel as an antioxidant supplementation in preserving semen in various animal species, which enhances the proportion of viable sperm. For instance, Baqir et al. [12] observed significant improvements in human sperm quality parameters with the administration of AV gel at a concentration of 5 µL/mL. Notably, a 20% *Aloe vera* concentration has been found to be a viable substitute for egg yolk in Tris extenders, promoting sperm motility in cooled semen of peccary [13]. Research by Agarwal et al. [14] revealed statistically significant enhancements in the quality of cattle sperm cells within the group supplemented with AV gel at a concentration of 5 µL/mL in the sperm extender when compared to the control group. However, the impact of *Aloe vera* supplementation as an antioxidant in rooster semen storage remains unexplored. Thus, the primary aim of the current study is to investigate the influence of *Aloe vera* gel supplementation in semen extenders on semen quality in native chickens by determining the optimal dosage, with a focus on evaluating fertility capacity. Additionally, the antioxidant properties of AV gel were determined.

## 2. Materials and Methods

### 2.1. Chemicals

Unless specified otherwise, all chemicals utilized in this research were purchased from Sigma-Aldrich Chemical Co. (St. Louis, MO, USA).

### 2.2. Aloe vera Gel Preparation

*Aloe vera* plants were harvested from Ban Fang District, Khon Kaen Province, located in northeastern Thailand. Only leaves from plants aged over four years were selected to ensure optimal quality and composition. The AV leaves were carefully harvested and rinsed with clean water to eliminate surface impurities. To prepare the gel, the thick outer skin of the leaves was peeled off to reveal the inner gel, which contains bioactive compounds. The inner gel was then cut into small pieces to facilitate extraction. The mucilage was separated by passing the gel pieces through a coarse sieve, followed by further filtration through a fine sieve to obtain a smooth crude extract. This filtration process is consistent with methods described in previous studies [13]. Once the crude extract of AV gel was obtained, it was securely stored in a freezer at −20 °C until required for use.

### 2.3. Antioxidant Property of AV Gel

The antioxidant properties of AV gel were determined by measuring its total phenolic content, total triterpenoid content, total polysaccharide content, and DPPH (2,2-diphenyl-1-picrylhydrazyl) radical scavenging activity before using it as an antioxidant supplement.

#### 2.3.1. Determination of Total Phenolic Content

The total phenolic content in the sample AV gel was determined using the modified Folin–Ciocalteu method, following the protocol described by Adebiyi et al. [15]. A gallic acid standard was prepared by dissolving gallic acid in distilled water at concentrations of 12.5, 25, 50, 100, 200, and 400 µg/mL. For the sample preparation, a concentration of 10 mg/mL was utilized in the reaction. In the reaction setup, 50 μL of the sample solution was combined with the Folin–Ciocalteu reagent in a 1:5 volume ratio, followed by the addition of 100 μL of a Na_2_CO_3_ solution (0.35 M or 75 g/L) in a 96-well plate or microplate. The mixture was then incubated for 30 min at room temperature. Subsequently, quantitative phenolic estimation was performed at 765 nm using a microplate reader (TECAN, infinite^®^ 200 PRO, Tecan Group Ltd., Männedorf, Switzerland), and the absorbance values were compared against a blank to determine the total phenolic content in the sample. A calibration curve was generated by plotting the absorbance against concentration, allowing the expression of total phenolic content in milligrams of gallic acid equivalent per gram of AV gel (mg GAE/g AV gel).

#### 2.3.2. Determination of Total Triterpenoid Content

The total triterpenoid content was determined following the methodology outlined by Chang et al. [16], utilizing ursolic acid as the standard reference. A sample aliquot of 100 μL, at a concentration of 10 mg/mL, was mixed with 150 μL of a vanillin–acetic acid solution (5% *w*/*v*) and 500 μL of a perchloric acid solution in a test tube. The solution was incubated at 60 °C for 45 min. After incubation, the test tube was transferred to a cold-water bath, and 2.25 μL of acetic acid was added. The mixture was then left at room temperature for 15 min. The absorbance was measured at a wavelength of 548 nm using a microplate reader. The absorbance values were compared to the ursolic acid standard curve to determine the total triterpenoid content. Total triterpenoid content was expressed as milligrams of ursolic acid equivalents per gram of AV gel (mg UA/g AV gel).

#### 2.3.3. Total Polysaccharide Content

The determination of total polysaccharide content followed the methodology described by Pawar and D’Mello [17]. To prepare the blank solution, 1 mL of distilled water was mixed with 1 mL of 5% phenol, followed by the addition of 5 mL of concentrated H_2_SO_4_. A stock solution of glucose at 100 µg/mL in distilled water was prepared for the standard and sample solutions. Subsequently, aliquots were drawn from this solution to achieve sugar concentrations within the 60 to 90 µg/mL range. Each sample solution was then combined with 1 mL of the 5% phenol solution, followed by the addition of 5 mL of concentrated H_2_SO_4_. After a 10 min incubation period, the absorbance was measured at 490 nm using a microplate reader against the prepared blank. The total polysaccharide content was calculated using a calibration curve generated from the standard glucose solution. The concentration of polysaccharides in the samples was determined from the linear equation of the calibration curve, typically represented as y = mx + b, where y is the absorbance, m is the slope of the curve, x is the concentration of glucose, and b is the y-intercept. The total polysaccharide content in the samples was expressed as milligrams of glucose equivalent per gram of sample (mg glucose/g AV gel).

#### 2.3.4. Antioxidant Capacity Measurement Using DPPH Assay

DPPH (2,2-diphenyl-1-picrylhydrazyl) is a free radical that is reduced when it encounters an antioxidant. Therefore, the DPPH assay is a widely used method to assess the free radical scavenging ability of potential antioxidants in various samples by determining the DPPH (%) inhibition as radical scavenging activity, following the method described by Xiao et al. [18]. Briefly, 100 μL of a 0.2 mM DPPH radical solution in ethanol was combined with 100 μL of AV gel solutions at varying concentrations. After a 30 min incubation period at room temperature in the dark, the absorbance was recorded at 517 nm using a microplate reader. Ascorbic acid was utilized as a positive control. The percentage inhibition of DPPH radicals was calculated using the formula Inhibition (%) = (A0 − A1)/A0 × 100, where A0 is the absorbance of the control (DPPH solution without the sample) and A1 is the absorbance of the sample. The AV gel’s IC50 value, the concentration required to inhibit 50% of the DPPH radicals, was determined from the inhibition curve.

### 2.4. Rooster Semen Collection, Dilution, and Semen Storage

In this study, sixty-four Thai native (Pradu Hang Dum) roosters, aged between 36 and 40 weeks, were used. These roosters were individually housed in battery cages within an open environment system. Their diet consisted of approximately 130 g of commercial feed (Balance 924, Betagro Company Limited, Bangkok, Thailand), providing 17% protein content. Semen samples from each rooster were collected twice weekly through dorso-abdominal massage and stored in a 1.5 mL microtube containing 100 µL of an IGGKPh extender [19]. The extender composition included 0.14 g potassium citrate, 1.40 g sodium glutamate, 0.21 g sodium dihydrogen phosphate, 0.98 g disodium hydrogen phosphate, 0.9 g glucose, and 0.9 g inositol in 100 mL of deionized water. The pH of the extender was maintained at 6.95, with an osmotic pressure of 380 mOsm/kg [19]. These samples were stored at a controlled temperature of 22–25 °C and transported to the laboratory within 30 min for the subsequent processing and analysis.

In the laboratory, the semen samples were evaluated, with mass movement assessed on a 0–5 scale under a compound microscope (100×). Sperm viability was determined using eosin–nigrosin staining. Only samples scoring a mass movement of ≥4 and sperm viability of ≥90% met the experimental criteria for further use.

Subsequently, the qualified semen samples were pooled and divided into treatment groups with varying concentrations of AV gel diluted in an IGGKPh extender at a ratio of 1:3 (*v*/*v*). The final concentration of the extended semen was maintained at approximately 100−150 × 10^6^ spermatozoa per dose [7]. The diluted semen was then cooled from 25 to 5 °C for 60 min and stored in a refrigerator at 5 °C for further processing and experimentation.

### 2.5. Experimental Design

To determine the optimal concentration of AV gel on sperm quality during storage for up to 72 h, the treatments were divided into eight groups based on varying levels of AV gel supplementation, 0% (control), 0.25%, 0.50%, 1.0%, 2.5%, 5.0%, 10%, and 20%, which were added to an IGGKPh extender. The diluted semen samples were subsequently stored at 5 °C. Sperm quality parameters in terms of sperm motility, viability, lipid peroxidation, and pH levels were assessed at 0, 24, 48, and 72 h of storage at 5 °C. The experiment was repeated eight times.

Fertility was evaluated using the treatment group, which demonstrated the most favorable outcomes after 24, 48, and 72 h of storage compared with the control group through artificial insemination in hens conducted once per week for a continuous period of 4 weeks. Fertility rates were recorded.

### 2.6. Evaluation of Sperm Quality

Sperm motility was assessed using a light microscope at a magnification of 400×. For the analysis, 5 µL of semen samples was diluted with 100 µL of the IGGKPh dilutor. Sperm motility was evaluated to determine the overall strength of sperm motility. The obtained data were used to calculate the percentage of motile sperm.

Sperm viability was assessed by the eosin–nigrosin staining technique [20]. A total of 10 μL of semen containing about 100 million sperm was combined with 100 μL of eosin–nigrosin dye at the same temperature as the semen. The dye and semen were mixed well and incubated for 3–5 min. Subsequently, drops of the stained semen were placed on slides, smeared, and allowed to dry. The evaluation of surviving sperm was conducted under a light microscope at 1000× magnification. Dead sperm were stained pink, while viable sperm remained unstained. Counting surviving sperm was carried out with a standardized count of 300 sperm in each sample. The data collected from these assessments were then utilized to calculate the percentage of normal sperm within the sample.

Lipid peroxidation was assessed by quantifying the quantity of malondialdehyde (MDA), a crucial marker indicative of oxidative damage, as it is the final product resulting from lipid peroxidation reactions. The evaluation method utilized thiobarbituric acid (TBA) as a test reagent, known for its ability to accurately measure MDA levels, as previously detailed in our study [21]. In the first assessment steps, semen samples were added with 0.25 mL of 1 mM ascorbic acid and 0.25 mL of 0.2 mM ferrous sulfate. Then, the samples were incubated at 37 °C for 60 min. Subsequently, a portion of the incubated semen samples underwent further processing by adding 1 mL of 15% (*w*/*v*) trichloroacetic acid and 1 mL of 0.375% (*w*/*v*) TBA. These samples were then boiled at 100 °C for 10 min in water. After the specified boiling duration was completed, the samples were rapidly cooled in ice water to halt the reaction between the reagent and the semen sample. Eventually, the samples were centrifuged at 1000× *g* for 10 min at 4 °C to separate the supernatants (approximately 2 mL) for the analysis. The MDA content in the semen samples was determined using a UV–visible spectrophotometer set at a wavelength of 532 nm (Analytik Jena Specord^®^ 250 plus, Jena, Germany).

The pH within each treatment group was measured using a pH meter (Crison Model Basic 20, Crison Instruments, S.A., Barcelona, Spain).

### 2.7. Fertility

For the fertility assessment, 48 commercial hens (Isa-brown) aged 29 weeks at the start of insemination, with an egg production of at least 70%, were randomly allocated into treatment groups. Throughout the experiment, every hen underwent weekly insemination sessions for four consecutive weeks. The insemination procedure involved a single intravaginal insemination of the layer hens on Day 0, with 0.1 mL of the respective sample administered to each group between 3:00 and 5:00 pm. The eggs were collected from Day 2 after the first insemination to the 8th day following the last insemination. These eggs were stored on paper trays within a controlled temperature environment ranging between 22 and 25 °C and were incubated weekly to ascertain fertility status on Day 7 (confirmed via candling). The fertility rate was calculated based on the total number of incubated eggs. The hatchability rate (total number of hatching eggs/total number of fertile eggs) was determined by the hatching of fertile eggs approximately 21 days after the start of incubation.

### 2.8. Statistical Analysis

The analysis of sperm quality data was performed using a split-plot design within a completely randomized design (CRD). The experimental treatments consisted of two factors where factor 1 included 8 treatments ranging from 0 (control) to various concentrations of AV gel at 0.25, 0.50, 1.0, 2.5, 5.0, 10, and 20%, while factor 2 represented the storage time of the semen extender at 4 intervals: 0 (after the cooling process), 24, 48, and 72 h (T0, T24, T48, and T72). The observation values included sperm motility, sperm viability, lipid peroxidation (MDA), and pH.

Regarding the fertility potential assessment, a split-plot design within a CRD was utilized for the analysis. Two factors were statistically analyzed: factor 1 included two treatments (control and AV gel at 1.0%), while factor 2 was insemination time after semen storage. The observation values were fertility and hatchability.

The statistical analysis was performed using ANOVA (Proc ANOVA), with the subsequent comparison of treatment groups using Tukey’s post hoc test. Statistical significance was considered at *p* < 0.05. The results were analyzed using the statistical software package SAS version 9.0.

## 3. Results

### 3.1. Bioactive and Antioxidant Capacity of Aloe vera Gel

Table 1 shows the bioactive and antioxidant capacity concentrations of *Aloe vera* (AV) gel. The results are presented as the mean ± standard deviation.

### 3.2. Effects of Different AV Gel Level Supplementation on Cooling Semen Quality during Storage

The interaction between storage time and treatment regarding sperm motility, sperm viability, lipid peroxidation, and pH exhibited a highly significant effect (*p* < 0.0001; Table 2), indicating a noteworthy impact of the varying levels of AV gel supplementation on the cooling semen quality during storage.

Table 3 shows the percentage of sperm motility, sperm viability, lipid peroxidation (MDA), and pH of semen diluted with an extender supplemented with different levels of AV gel and stored for 72 h at 5 °C. Sperm quality, in terms of sperm motility and sperm viability, was highly significant with 1.0% AV gel supplementation (*p* < 0.05) across all storage times. However, sperm decreased as storage time increased (*p* < 0.05), regardless of the treatment. Notably, in the 1.0% AV gel group, the sperm quality in terms of sperm motility and sperm viability for storage at 24 h (T24) (85.54% and 95.50%, respectively) was not significantly different from those at 0 h (T0) (85.67% vs. 92.46%, respectively). Conversely, the supplementation of the semen extender with higher levels of AV gel starting from 2.5% and above had an increasingly detrimental effect on sperm quality, with the lowest sperm quality observed in the 20% AV gel group across all storage times (*p* < 0.05).

The results of lipid peroxidation, as presented by MDA, demonstrated that significantly lower MDA levels were observed in the group supplemented with 0.25–1.0% AV gel compared to the other AV groups and the control group at all storage times (*p* < 0.05). Among these, the lowest MDA levels within the 1.0% AV gel group were found at 0 h and 72 h after storage.

The pH values did not show any statistical differences when comparing among treatment groups at T0 (*p* > 0.05). However, significant differences in pH were observed in the groups supplemented with AV gel at concentrations starting from 2.5% and above, with the lowest pH values recorded from T24 to T72 after storage (*p* < 0.05).

Based on the results related to sperm quality, lipid peroxidation, and pH, the AV gel at a concentration of 1.0% was chosen for further investigation into fertility.

### 3.3. Effects of the Best Extender on Fertility and Hatchability

The fertility potential results, when comparing semen diluted with 1.0% AV gel to the control group, are presented in Table 4. The data indicate that both storage duration and treatment significantly influence fertility and hatchability rates. The storage duration of semen has a significant impact on fertility potential, with fertility ability decreasing as the storage time increases (*p* < 0.05). However, the fertility and hatchability rates were consistently better in the group inseminated with semen diluted with 1.0% AV gel compared to the control group at all evaluation periods.

## 4. Discussion

Preserving rooster semen presents some challenges compared to other species due to the unique characteristics of avian sperm. Rooster semen has a relatively short lifespan, which makes it crucial to process and store it promptly after collection. Additionally, rooster sperm is highly susceptible to damage during preservation, which involves numerous sources of oxidative stress from dilution, limiting its viability and fertility ability to less than 24 h under cool preservation conditions. Therefore, researchers are continuously working on optimizing preservation methods to enhance the viability and longevity of rooster semen. In the present study, the supplementation of the extender with 1.0% AV gel provided a beneficial antioxidant substance by decreasing lipid peroxidation and concurrently improving sperm quality during cool preservation at 5 °C for up to 72 h. Although an increase in MDA levels in cooled semen was observed as storage time increased, fertility capacity remained higher and was maintained at over 60% for up to 72 h. Additionally, it might be noted that higher doses of AV gel supplementation negatively affect sperm quality despite having greater antioxidant capacity, indicating that optimal dosage levels are necessary to achieve desired improvements in semen quality during cold storage.

*Aloe vera* is recognized for its therapeutic properties, including its potent antioxidant property [22]. This antioxidant capacity stems from its content of phenolic compounds, which contribute to energy production and membrane stability in sperm cells [23,24]. Moreover, *Aloe vera* is rich in polysaccharides [25], which further contribute to its antioxidant effects by protecting sperm from free radical damage [26]. These polysaccharides have also been linked to enhanced sperm quality [27]. The AV gel used in this study exhibited significant total phenolic content and antioxidant capacity, as well as the presence of polysaccharides (Table 1). These findings support the hypothesis that AV gel supplementation could effectively protect rooster sperm during cool preservation. Our results (Table 3) demonstrate that the 1.0% AV gel treatment group exhibited a significant reduction in lipid peroxidation and improvements in sperm motility and viability compared to other groups during 72 h of storage. This enhanced sperm quality aligns with previous research by Agarwal et al. [14] and Zareie et al. [24], who also reported significant improvements in sperm quality with AV gel supplementation in extenders.

While low-temperature cold preservation slows down sperm metabolic activity, it does not eliminate it entirely. Anaerobic respiration and free radical accumulation still occur during storage, leading to lactic acid production and a decrease in semen pH [20]. This decrease in pH of the semen diluent as storage time increases, as observed in our study, is a known factor that can negatively impact sperm motility. Furthermore, the absence of antibiotics in our extender resulted in bacterial contamination originating from the cloaca. Our preliminary investigation recorded a notable bacterial count, reaching as high as 135 × 10^2^ CFU/mL, with no significant decrease over time (approximately 127 × 10^2^ CFU/mL at 72 h of storage). These bacteria contribute to the further metabolic activity and acidification of the semen [28], compounding the challenges of long-term preservation.

Another noteworthy finding in our experiment was a significant decrease in pH with increasing AV gel supplementation in semen extenders. This pH reduction is likely attributed to the high polysaccharide content in the AV gel. While polysaccharides offer protection against oxidative stress, their breakdown can produce acid compounds, such as acetic acid and gluconolactone [25,29], contributing to a lower pH. Previous research suggested that high concentrations of AV gel may have detrimental effects on male reproductive performance. For instance, studies have shown that feeding roosters 3–5% AV gel in water for 14 days resulted in abnormal sperm percentages [30]. Similarly, Nesslany et al. [31] observed toxicity to the reproductive system of mice when administered 500, 1000, and 2000 mg/kg of AV leaf extracts. Furthermore, Golestan et al. [32] reported increased MDA levels and reduced resilience in rainbow trout following supplementation with 0.5, 1, and 2 g/kg of *Aloe vera* in fish feed. These findings indicate that the potential toxicity of AV gel at higher concentrations warrants careful consideration. While AV gel at lower concentrations (1.0% in our study) showed beneficial effects on sperm quality, further investigation is necessary to optimize the dosage and minimize any adverse effects on sperm preservation and overall reproductive performance.

Fertility potential is a critical factor in the reproductive process. While limited information exists on the viability of cold-stored semen beyond 24 h, our study aimed to assess fertility using stored semen for up to 72 h. A recent study by Kheawkanha et al. [33] conducted fertility assessments using solid semen for up to 120 h after storage and demonstrated a decline in fertility, reaching only approximately 50% at that storage duration. This decline may be attributed to decreased sperm motility, which can hinder sperm deposition in the sperm storage tubules (SSTs) for fertilization [9]. Our study, however, yielded promising results. The fertility rates of semen stored for up to 72 h using our extender (Table 4) were consistently higher than the control group at the same storage duration. Notably, the fertility rate of the treatment group at T48 (77.64%) was comparable to that of the control group at T24 (78.53%). These findings exceed the reported fertility rates in previous studies, such as those using creatine supplementation in Vietnamese native chickens (maximum 60% fertility at 48 h of storage) [34] and northern pintail (approximately 20% fertility at 72 h) [35].

While this study demonstrated high fertility rates, the observed hatchability was lower than expected. This suggests that factors impacting embryo development, rather than initial fertilization, may be contributing to the lower hatchability. Several factors, including hen age and heat stress, can influence egg quality and embryo development. Uni et al. [36] showed that hen age significantly affected egg size, yolk, and albumen components, with larger eggs exhibiting higher hatchability. In this study, the hens were 29 weeks old, which is an early laying period associated with smaller eggs, potentially contributing to the lower hatchability. Temperature and photoperiod also play crucial roles in poultry egg fertility and hatchability [37]. High temperatures, or heat stress, can negatively impact egg production and shell quality due to physiological responses such as hyperventilation and reduced calcium deposition [38]. Laying hens under heat stress may produce eggs with lower quality due to imbalances in calcium–estrogen relationships and decreased feed intake [38]. Our previous research [39] indicated that Thailand experienced its highest temperature–humidity index values during the AI program period (March–April). This finding aligns with Suwimonteerabutr et al. [40], who also reported significant heat stress in breeder hens from February–May in Thailand. Maintaining optimal environmental conditions, especially temperature and humidity, is crucial for maximizing fertility and hatchability in poultry.

This study focused on the antioxidant properties of AV gel as a semen supplement for sperm preservation; it has several limitations that warrant further investigation. Firstly, the beneficial effects of AV gel on sperm preservation might involve a complex interplay of various bioactive components. Research has demonstrated that amino acids (e.g., GABA, Alanine, and Arginine) and fatty acids (e.g., linolenic acid and palmitic acid) found in AV gel exhibit antioxidant activity [41,42,43]. These components can protect sperm from oxidative stress, a major factor contributing to sperm damage and infertility. Future research should specifically investigate the effects of these components on sperm parameters (motility, morphology, DNA integrity) to fully elucidate their potential for enhancing sperm preservation techniques. Secondly, the use of fresh AV gel without solvent extraction likely resulted in a lower concentration of certain bioactive compounds compared to studies employing extraction methods [44,45]. Different solvents can extract distinct phytochemical profiles, with ethanol and methanol often yielding higher antioxidant activities [46,47]. The extraction method and solvent used can significantly influence the yield of bioactive compounds and the overall antioxidant potential of *Aloe vera* extracts [48]. While our study provides a natural assessment of *Aloe vera* gel’s properties, future research exploring different solvent systems and extraction techniques would be necessary to fully understand its antioxidant potential and its applications in sperm preservation.

## 5. Conclusions

This study demonstrates the potential of supplementing a semen extender with 1.0% AV gel for enhancing the preservation of Thai native rooster semen at 5 °C for up to 72 h. Our findings suggest that this approach effectively mitigates oxidative stress, improves sperm quality, and maintains high fertility rates compared to traditional methods. However, it is important to note that this study has several limitations. Future research investigating other potential bioactive components in terms of amino and fatty acids, also exploring different solvent systems and extraction techniques, would be necessary to fully understand its antioxidant potential and its applications in sperm preservation. Despite these limitations, this research provides a promising avenue for improving rooster semen preservation techniques, which could significantly benefit the poultry industry.

## Figures and Tables

**Table 1:** Concentrations of the bioactive properties and antioxidant capacity of AV gel.

Compound	Content
Total phenolic (mg GAE/g)	1.73 ± 0.04
Total polysaccharide (mg glucose/g)	359.33 ± 4.19
Total triterpenoid (mg UA/g)	18.51 ± 1.18
Antioxidant capacity (DPPH (%) inhibition)	17.46 ± 0.44

**Table 2:** Summary of analysis of variance results for sperm motility, sperm viability, lipid peroxidation (MDA), and pH of semen diluted with extender supplementing different levels of AV gel and stored for 72 h at 5 °C.

Source of Variation	Level of Significance (*p*-Value)		
	Time of Storage	Treatment	Time of Storage × Treatment
Sperm motility	<0.0001	<0.0001	<0.0001
Sperm viability	<0.0001	<0.0001	<0.0001
MDA	<0.0001	<0.0001	<0.0001
pH	<0.0001	<0.0001	<0.0001

**Table 3:** Percentage of total motility, viability, lipid peroxidation (MDA), and pH of semen diluted with extender supplementing different levels of AV gel and stored for 72 h at 5 °C.

Time of Storage (h)	Treatments								SEM
	Control	0.25%	0.50%	1.0%	2.5%	5.0%	10%	20%	
Sperm motility									
T0	85.67 ^cw^	89.37 ^bw^	94.83 ^aw^	95.88 ^aw^	82.21 ^dw^	78.54 ^ew^	72.17 ^fw^	65.88 ^gw^	0.70
T24	80.54 ^dx^	88.75 ^ax^	83.21 ^cx^	85.54 ^bx^	74.97 ^ex^	74.43 ^ex^	69.75 ^fx^	55.63 ^gw^	
T48	71.60 ^cy^	74.24 ^by^	75.62 ^by^	79.00 ^ay^	72.54 ^cy^	70.67 ^dy^	62.29 ^ey^	42.62 ^fy^	
T72	65.62 ^dz^	71.79 ^cz^	74.42 ^bz^	77.88 ^az^	61.92 ^ez^	57.87 ^fz^	51.13 ^gz^	32.54 ^hz^	
Sperm viability									
T0	92.46 ^cw^	95.67 ^bw^	96.2 ^bw^	98.83 ^aw^	91.33 ^cw^	82.96 ^dw^	74.04 ^ew^	72.33 ^fw^	0.53
T24	85.21 ^cx^	90.67 ^bx^	92.42 ^bx^	95.50 ^ax^	81.42 ^dx^	77.83 ^ex^	77.63 ^ex^	62.54 ^fx^	
T48	74.68 ^cy^	77.29 ^by^	79.17 ^ay^	80.67 ^ay^	74.08 ^cy^	72.63 ^dy^	63.92 ^ey^	45.75 ^fy^	
T72	71.88 ^cz^	75.33 ^bz^	75.97 ^bz^	79.17 ^az^	74.88 ^bz^	62.46 ^dz^	55.04 ^ez^	34.13 ^fz^	
MDA									
T0	1.09 ^dz^	0.68 ^ez^	0.59 ^gz^	0.54 ^hz^	0.62 ^fz^	1.15 ^cz^	1.24 ^bz^	1.35 ^az^	0.02
T24	2.14 ^dy^	1.09 ^fy^	1.09 ^fy^	1.09 ^fy^	2.07 ^ey^	3.33 ^cy^	3.56 ^by^	3.79 ^ay^	
T48	4.23 ^cx^	3.09 ^ex^	3.06 ^ex^	3.04 ^ex^	4.06 ^dx^	5.57 ^bx^	5.90 ^ax^	5.92 ^ax^	
T72	5.73 ^dw^	5.49 ^ew^	5.35 ^fw^	5.26 ^gw^	5.34 ^fw^	5.94 ^cw^	7.33 ^bw^	7.66 ^aw^	
pH									
T0	7.00 ^aw^	7.00 ^aw^	7.00 ^aw^	7.00 ^aw^	6.99 ^abw^	6.99 ^abw^	6.98 ^abw^	6.98 ^abw^	0.04
T24	6.99 ^abx^	6.99 ^abx^	6.99 ^abx^	6.99 ^abx^	6.95 ^cx^	6.89 ^dx^	6.84 ^ex^	6.82 ^efx^	
T48	6.94 ^ay^	6.94 ^ay^	6.93 ^aby^	6.93 ^aby^	6.88 ^ey^	6.81 ^fy^	6.74 ^hy^	6.73 ^hy^	
T72	6.91 ^az^	6.90 ^abz^	6.90 ^abz^	6.90 ^abz^	6.84 ^dx^	6.72 ^fz^	6.63 ^hz^	6.61 ^hz^	

SEM, standard error of the means. ^a, b, c, d, e, f, g, h^ (different letters within a row) and ^w, x, y, z^ (different letters within a column) indicate the interaction between treatment effects and time to storage effect significant differences (*p* < 0.05).

**Table 4:** Effects of different extenders with different times to storage on the percentages of fertility and hatchability after artificial insemination using semen diluted with 1.0% AV gel compared to the control group.

Time to Storage (h)	Treatments		SEM	*p*-Values		
	Control	1.0%		Treatments ^1^	Time to Storage ^2^	Interaction
No. of eggs	459	474				
Fertility						
T24	78.53 ^bx^	83.36 ^ax^	3.75	<0.0001	<0.0001	0.2316
T48	74.67 ^by^	77.64 ^ay^				
T72	65.29 ^bz^	69.05 ^az^				
Hatchability						
T24	64.31 ^bx^	72.33 ^ax^	1.86	<0.0001	<0.0001	0.0560
T48	61.58 ^by^	68.84 ^ay^				
T72	55.21 ^bz^	59.26 ^az^				

^1.^ For treatment effects, means within a row with superscript letters a and b indicate significant differences (*p* < 0.05). ^2.^ For time-to-storage effects, means within a column with superscript x, y, and z indicate significant differences (*p* < 0.05).

## Data Availability

The data are available upon request to the corresponding author.
